# Role of *Hemigraphis alternata* in wound healing: metabolomic profiling and molecular insights into mechanisms

**DOI:** 10.1038/s41598-024-54352-x

**Published:** 2024-02-16

**Authors:** Rex Devasahayam Arokia Balaya, Akhina Palollathil, Sumaithangi Thattai Arun Kumar, Jaikanth Chandrasekaran, Shubham Sukerndeo Upadhyay, Sakshi Sanjay Parate, M. Sajida, Gayathree Karthikkeyan, Thottethodi Subrahmanya Keshava Prasad

**Affiliations:** 1 Center for Systems Biology and Molecular Medicine, Yenepoya Research Centre, Yenepoya (Deemed to be University), Mangalore, India 575018; 2 Department of Pharmacology, Sri Ramachandra Faculty of Pharmacy, Sri Ramachandra Institute of Higher Education and Research (Deemed to be University), Chennai, 600116 India; 3 Yenepoya Research Centre, Yenepoya (Deemed to be University), Mangalore, India; 4 Present Address: Department of Laboratory Medicine and Pathology, Mayo Clinic, Rochester, MN USA

**Keywords:** Mass spectrometry, Metabolomics

## Abstract

*Hemigraphis alternata* (*H. alternata*), commonly known as Red Flame Ivy, is widely recognized for its wound healing capabilities. However, the pharmacologically active plant components and their mechanisms of action in wound healing are yet to be determined. This study presents the mass spectrometry-based global metabolite profiling of aqueous and ethanolic extract of *H. alternata* leaves. The analysis identified 2285 metabolites from 24,203 spectra obtained in both positive and negative polarities. The identified metabolites were classified under ketones, carboxylic acids, primary aliphatic amines, steroids and steroid derivatives. We performed network pharmacology analysis to explore metabolite–protein interactions and identified 124 human proteins as targets for *H. alternata* metabolites. Among these, several of them were implicated in wound healing including prothrombin (F2), alpha-2A adrenergic receptor (ADRA2A) and fibroblast growth factor receptor 1 (FGFR1). Gene ontology analysis of target proteins enriched cellular functions related to glucose metabolic process, platelet activation, membrane organization and response to wounding. Additionally, pathway enrichment analysis revealed potential molecular network involved in wound healing. Moreover, in-silico docking analysis showed strong binding energy between *H. alternata* metabolites with identified protein targets (F2 and PTPN11). Furthermore, the key metabolites involved in wound healing were further validated by multiple reaction monitoring-based targeted analysis.

## Introduction

*Hemigraphis alternata* (*H. alternata*) belongs to the Acanthaceae family and is distributed in the eastern Malaysia region and adapted to India^1,2^. It is commonly known as red flame ivy, purple waffle plant, and Murikooti. *H. alternata* is an annual creeping perennial herb growing up to 30 cm tall. The leaves are simple with a toothed margin and arranged in the opposite direction. The leaf blades are silver-coloured on the top and reddish-brown on the bottom, and the stem is erect and reddish-brown. Historically, the leaves of this plant have been used for wound healing and to treat anemia, hemorrhage, dysentery, and kidney stones^3^. In addition, leaf extract can be applied to fresh cuts to help them cure quickly^4^. *H. alternata* is reported to contain many phytoconstituents such as alkaloids, flavonoids, carbohydrates, steroids, triterpenes, phenols and amino acids^5^. A few studies have reported the antimicrobial, antioxidant, analgesic, and anti-inflammatory activities of *H. alternata*. Further, an in-silico docking study revealed a higher affinity of compounds from *H. alternata* for COX-1 (pharmacological targets for anti-inflammatory drugs) compared to the drug aspirin^6^. Moreover, *H. alternata* also possesses hepato-protective, nephroprotective, anti-ulcer, and anti-diabetic effects^1,7–9^. Recent research has indicated that *H. alternata* has promising potential for application in the field of biomedicine, particularly as an effective agent for wound healing. A biodegradable biofilm developed from agar/pectin composite containing *H. alternata* extract showed increased anti-microbial, hydrophilic and anticancer properties^10^. Further, a nanocellulose composite blended with *H. alternata* extract showed effective anti-microbial properties against diverse bacteria suggesting its potential as a wound dressing material^11^. Another study by Sasidharan et al*.* reported that glod nanoparticle-aided skin substitutes embedded with extracts of *Cyperus rotundus* and *H. alternata* have effective anti-microbial and anti-fungal activities^12^.

A wound is a break in the integrity of the epithelium, accompanied by a disruption in the cellular, anatomical and functional continuity of living tissue and can be caused by physical, chemical, thermal, microbial or immunological abuse^13^. In the setting of skin injury, the healing of a wound, following hemostasis, occurs in three overlapping stages: inflammation, proliferation and remodeling^14^. The wound-healing process is improved by shortening the time needed for healing or lowering the amount of incorrect healing, via administering drugs locally or systemically^15^. Antibiotics, antiseptics, and plant extracts have been used to achieve this^16,17^.

Medicines derived from local plants are used by several cultures and ethnic groups to heal wounds. Metabolites from medicinal plants such as *Moringa oleifera*, *Azadirachta indica, Glycyrrhiza glabra,* and others have been used for the treatment of incision and acute/chronic wounds^18–20^. Shedoeva et al*.*^21^ have described the wound-healing properties, active compounds, and clinical value of 36 medical plants including *Centella asiatica, Curcuma longa*, *Ampelopsis japonica, Ganoderma lucidum and Paeonia suffruticosa*. Extracts from the aerial parts of *Centella asiatica* facilitate the wound healing process in both incision and burn wounds by improving the angiogenesis and anti-inflammatory effect^22,23^. Curcumin has been shown to fasten wound healing because of its anti-inflammatory, anti-oxidant, anti-coagulant and anti-infective effects^24^.

There is an increasing interest in alternative medicinal plants for wound healing due to lower side effects and wound management over the years. A few bioactive compounds and their potential mechanisms have been reported to improve wound healing by medicinal plants^21,25,26^. Conducting a global metabolite profiling of alternative medicinal plants implicated in wound healing can unveil a network of various bioactive compounds linked to this process^27^. In this regard, by using a liquid chromatography-tandem mass spectrometry (LC–MS/MS)-based global metabolomics approach, we analyzed the metabolite profile of the *H. alternata* leaf extract and followed a network pharmacology approach to decipher potential molecular action to bring about its wound-healing ability. We believe this dataset will pave the way for further translational clinical research on wound healing.

## Results

### LC–MS/MS analysis of *H. alternata* leaf extracts

#### Identification of *H. alternata* metabolites at the MS1 level

**Figure 1:**
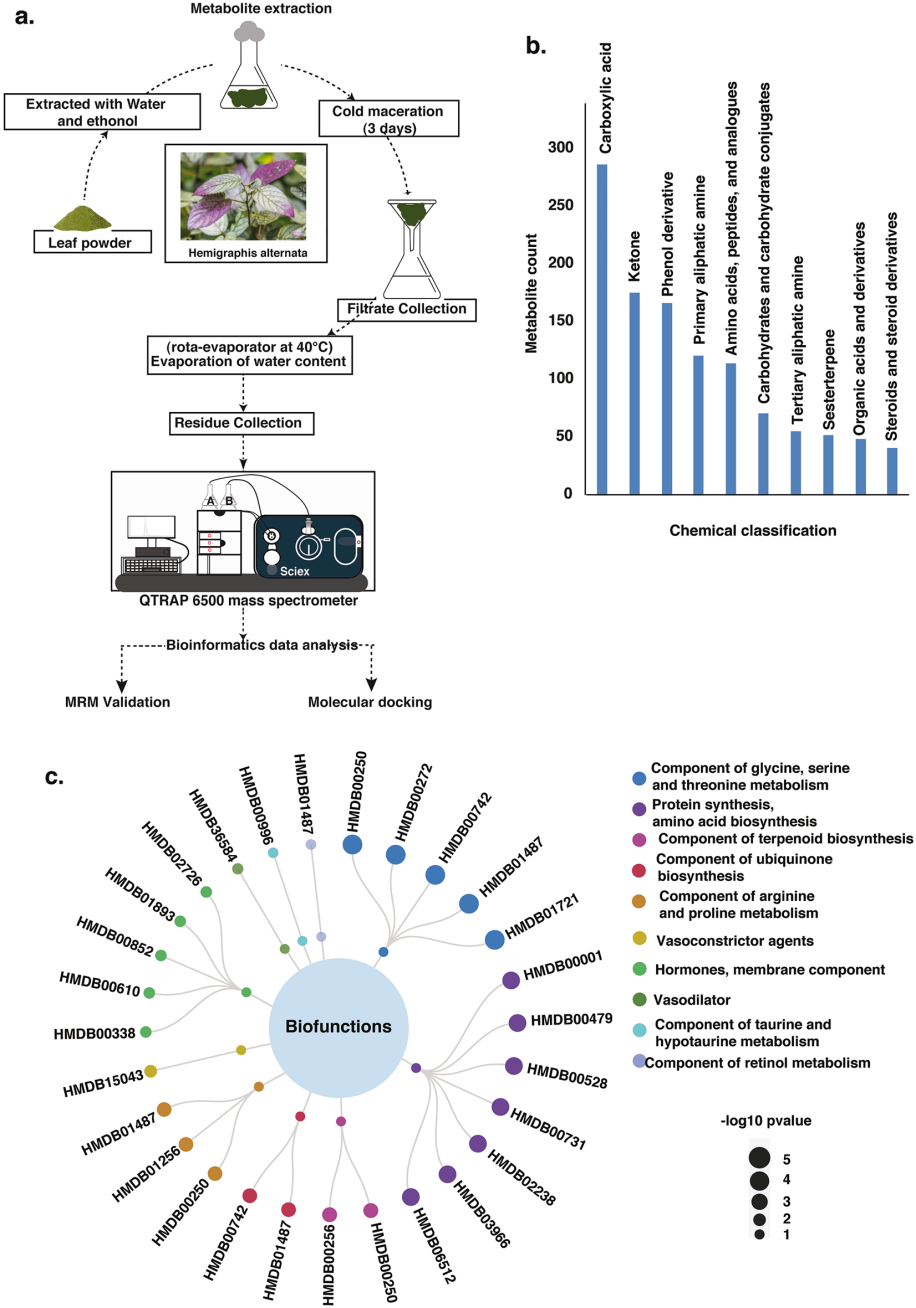
Biological classification of metabolites identified from *H. alternata.* (**a**) Methodolohy workflow employed metabolomics profiling and downstream analysis. (**b**) Bar diagram depicting the top 10 enriched metabolite classes along with the metabolites count. (**c**) Represents the highly enriched biofunctions of metabolites identified from *H. alternata.*

*H. alternata* is a medicinal plant used for healing wounds and to treat anemia and hemorrhage. We carried out the LC–MS/MS analysis of an aqueous and ethanolic extract of *H. alternata* to identify the global metabolite profile and to determine the metabolites involved in wound healing (Fig. 1a). This resulted in the identification of 24,203 spectra from both positive and negative polarities, which corresponds to 2285 metabolites. Metabolite identification at the MS1 level was carried out using MZmine as well as the XCMS Online tool^28^. The metabolites identified from MZmine analysis of aqueous extract included 708 non-redundant metabolites (335 in negative mode and 402 in positive mode) (Supplementary Table S1) and XCMS analysis identified 604 non-redundant metabolites (352 in negative mode and 267 in positive mode) from (Supplementary Table S2).

The metabolites identified from ethanolic extract included 685 non-redundant metabolites (322 in negative mode and 383 in positive mode) from MZmine analysis (Supplementary Table S3) and 548 non-redundant metabolites (325 in negative mode and 236 in positive mode) from XCMS analysis (Supplementary Table S4). MZmine and XCMS Online analysis resulted in the identification of 138 common metabolites and a total of 1157 and 735 metabolites were found unique to MZmine and XCMS Online analysis (Supplementary Table S5).

### Identification of *H. alternata* metabolites at MS/MS level

Metabolite fragment features obtained from MZmine analysis were used to assign metabolites at the MS/MS level with the help of the MS2Compound tool^29^. In the aqueous extract, 250 non-redundant metabolites were assigned at MS/MS level (90 in negative mode and 165 in positive mode) (Supplementary Table S6), whereas in the ethanolic extract, 265 non-redundant metabolites were assigned (117 in negative mode and 149 in positive mode) (Supplementary Table S7). Among the metabolites identified at the MS1 level, 57 metabolites showed MS/MS levels identification (Supplementary Table S8).

### Functional enrichment analysis of *H. alternata* metabolites

The non-redundant metabolites identified from ethanolic and aqueous extract were used to assess their chemical class and biofunctions using the MBROLE tool, and *Arabidopsis thaliana* was used as a reference database^30^. The chemical classification shows the enrichment of classes including ketones, phenol derivatives, steroids, and carboxylic acids to name a few. The top 10 classes and number of metabolites involved were represented in Fig. 1b, a detailed list is provided in Supplementary Table S9. The highly enriched biofunctions include the component of glycine, serine and threonine metabolism, protein synthesis, component of terpenoid biosynthesis, the component of arginine and proline metabolism, and vasoconstrictor agents (Fig. 1c).

**Figure 2:**
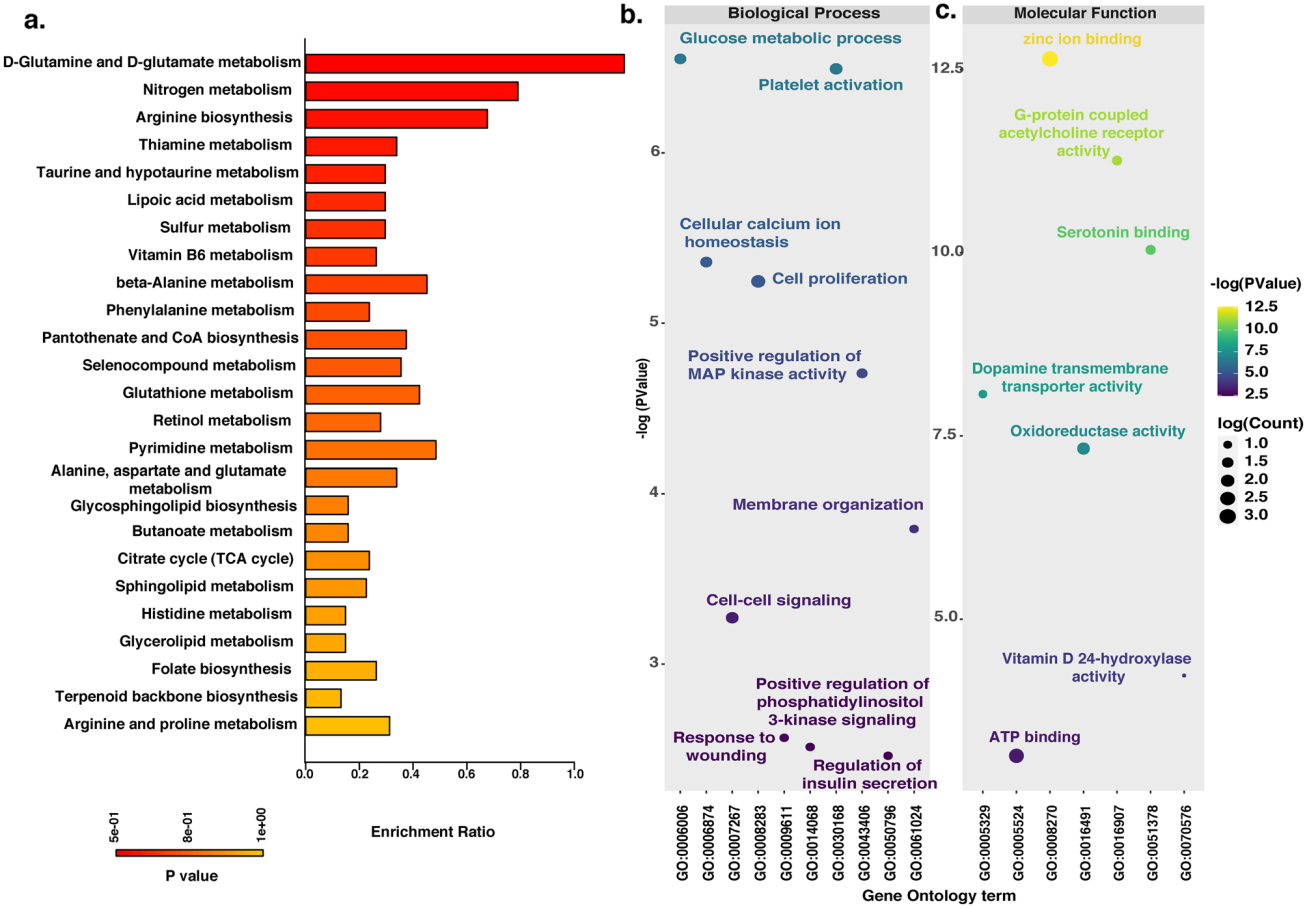
Functional enrichment of metabolites identified from *H. alternata.* (**a**) The pathway enrichment analysis for metabolites identified in aqueous and ethanolic extract of *H. alternata*. The X-axis represents the enrichment ratio and the Y-axis represents the pathways. (**b**) Gene Ontology analysis of protein targets of *H. alternata* highlighting the enriched biological processes, and (**c)** molecular functions. The X-axis represents the Gene Ontology terms and the Y-axis represents the − log (*p* value).

Pathway analysis using the MetaboAnalyst tool shows that *H. alternata* metabolites were involved in pathways of D-glutamine and D-glutamate metabolism, thiamine metabolism, taurine and hypotaurine metabolism, pyrimidine metabolism, alanine, aspartate, glutamate metabolism, glutathione metabolism, arginine and proline metabolism, and sphingolipid metabolism. Figure 2a represents the major pathways along with their enrichment ratio and *p*-value.

### Protein targets of *H. alternata* metabolites

Metabolites serve various biological functions as cofactors, allosteric regulators, and protein-complex assembly mediators and influence various biological pathways by interacting with proteins. Tracking the protein-metabolite interaction will help to understand the possible biological role of metabolites. The human protein targets of *H. alternata* metabolites were predicted by performing BindingDB analysis hoping to discover biologically active network of metabolites associated with wound healing. We identified 124 protein targets, which are implicated in wound-healing activity (Supplementary Table S10). To get more insight into the biological functions of these proteins, further analysis was carried out.

### Gene Ontology analysis of targets protein of *H. alternata* metabolites

Gene Ontology (GO) analysis was carried out for the protein targets of *H. alternata* metabolites to identify their biological processes and molecular functions using DAVID software. Based on biological processes, the protein targets were mainly involved in wound healing processes such as cell proliferation, platelet activation, cellular calcium ion homeostasis, membrane organization, positive regulation of phosphatidylinositol 3-kinase signaling, glucose metabolic process, and response to wounding (Fig. 2b). The protein targets were involved in molecular functions including oxidoreductase activity, zinc ion binding, dopamine transmembrane transporter activity, ATP binding, and G-protein coupled acetylcholine receptor activity (Fig. 2c).

### Pathway analysis of protein targets of *H. alternata* metabolites

**Figure 3:**
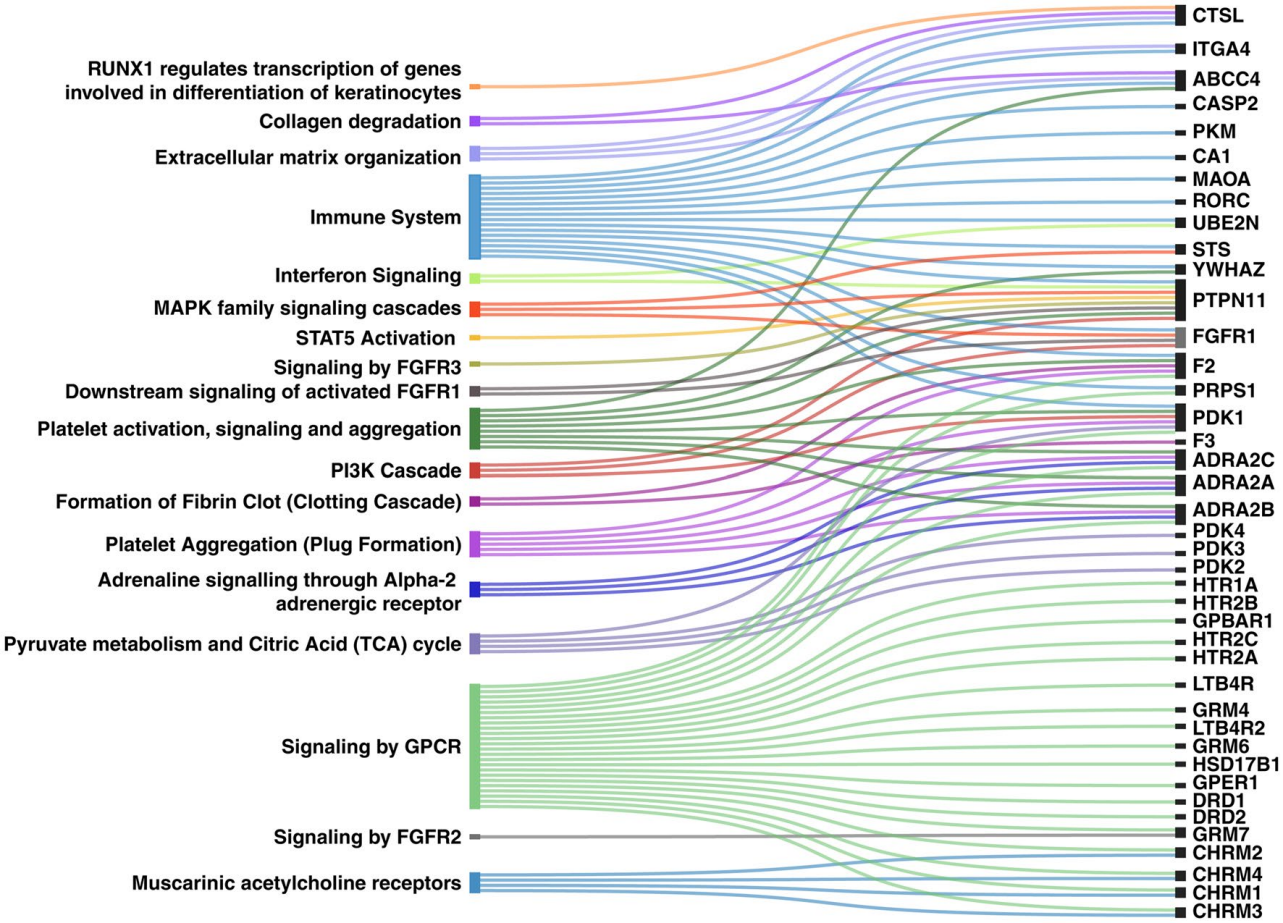
The Sankey diagram illustrating the enriched pathways associated with protein targets of *H. alternata* metabolites and corresponding target genes.

We performed pathway analysis by using the Reactome pathway analysis database to understand the biological pathways regulated by protein targets of *H. alternata* metabolites. The results show the enrichment of target proteins in pathways of platelet activation, signaling and aggregation (*ABCC4, PTPN11, ADRA2C, F2, ADRA2B, YWHAZ, ADRA2A, PDK1*), platelet aggregation (plug formation) (*ADRA2C, F2, ADRA2B, ADRA2A, PDK1*), PI3K cascade (*PTPN11, FGFR1, PDK1*), adrenaline signaling through Alpha-2 adrenergic receptor in the wound healing process (*ADRA2C, ADRA2B, ADRA2A*), *RUNX1* regulates transcription of genes involved in differentiation of keratinocytes and downstream signaling of activated FGFR1 (Fig. 3).

### Molecular docking analysis

In this study, we conducted a comprehensive molecular docking analysis to identify *H. alternata* metabolites involved in the coagulation cascade, focusing particularly on their interactions with essential proteins involved in wound healing pathways. To delve deeper into the potential biological activities of *H. alternata* metabolites towards their protein targets, we employed an in-depth docking analysis, centering on two crucial wound healing regulators: F2 and PTPN11. Utilizing the Glide 7.7 module of the Schrödinger suite, our study identified a significant active site within the F2/Prethrombin-2 complex (Supplementary Fig. S1). While the technique of active site analysis is well-established, our specific focus on this particular complex and the subsequent docking of *H. alternata* metabolites provided a unique perspective in our research. The top-ranked site within this complex, which achieved a Site Score of 1.04, was strategically situated at the interface of the F2/Prethrombin-2 complex and served as a pivotal point for our docking study.

**Figure 4:**
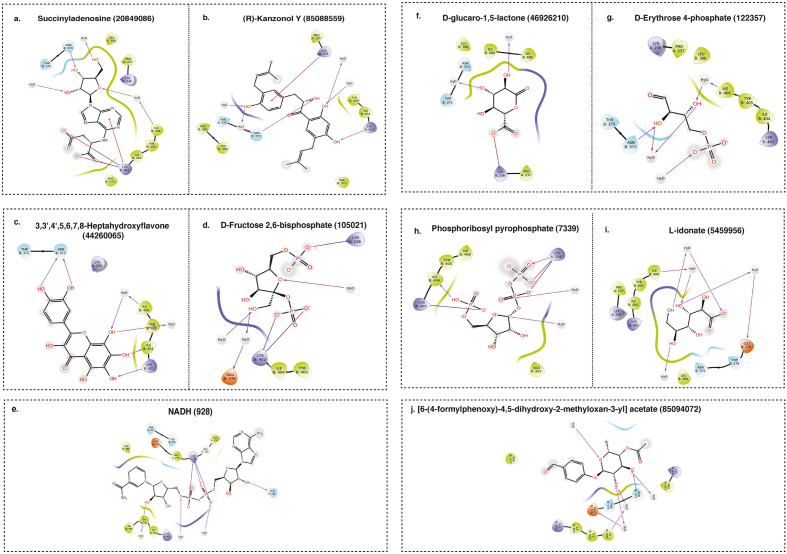
Molecular docking interaction between metabolites and proteins. Docking Poses and interaction patterns of the top hit 5 metabolites from (**a–e)** aqueous and (**f–j)** ethanolic extract of *H. alternata* with Prothrombin (F2).

In this process, we docked a total of 515 metabolites (264 from the aqueous extract and 251 from the ethanolic extract) revealing a range of binding affinities. The aqueous extract metabolites exhibiting high binding affinity towards F2-Prothrombin included Succinyladenosine (PubChem CID: 20849086), (R)-Kanzonol Y (PubChem CID: 85088559), 3,3′,4′,5,6,7,8-Heptahydroxyflavone (PubChem CID: 44260065), D-Fructose 2,6-bisphosphate (PubChem CID: 105021), and NADH (PubChem CID: 928), with docking scores of − 5.6, − 5.2, − 5.4, 5.9, and − 7.3 kcal/mol, respectively (Fig. 4a–e). Moreover, from the ethanolic extract, metabolites such as D-glucaro-1,5-lactone (PubChem CID: 46926210), D-Erythrose 4-phosphate (PubChem CID: 122357), Phosphoribosyl pyrophosphate (PubChem CID: 7339), L-idonate (PubChem CID: 5459956), and [6-(4-formylphenoxy)-4,5-dihydroxy-2-methyloxan-3-yl] acetate (PubChem CID: 85094072) also demonstrated significant binding affinity toward F2-Prothrombin, with docking scores of − 5.1, − 5.7, − 7.2, − 7.3, and − 5.26 kcal/mol, respectively (Fig. 4f–j).

**Figure 5:**
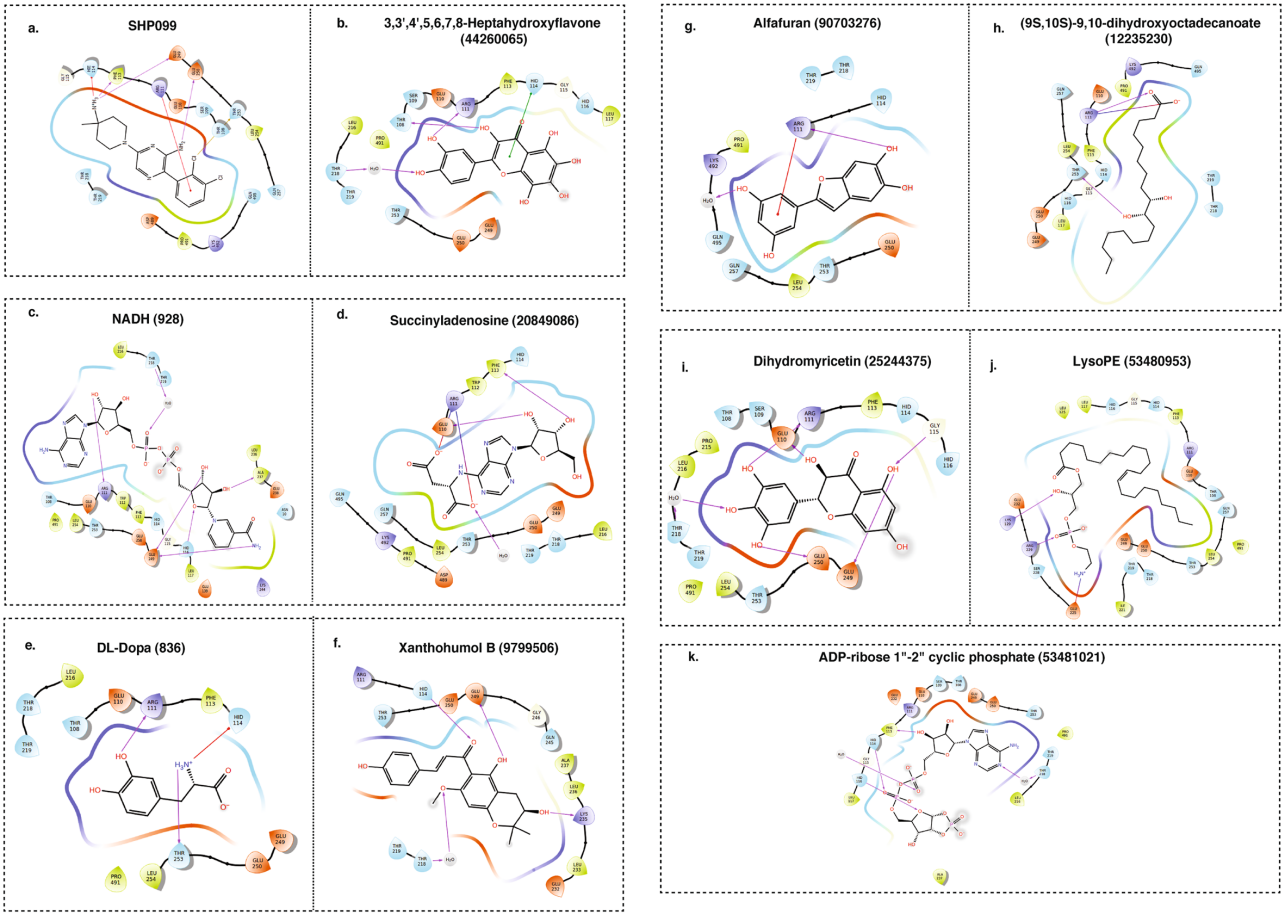
Molecular docking interaction of metabolites with proteins. Docking Poses and interaction patterns of the top hit 5 metabolites from (**a–e)** aqueous and (**e–j)** ethanolic extract of *H. alternata* with PTPN11.

Likewise, in the exploration of Tyrosine-protein phosphatase non-receptor type 11 (PTPN11), we utilized the protein structure 5EHR from the Protein Data Bank (PDB), complexed with the potent inhibitor SHP099. This foundational analysis enabled us to discern key interaction patterns, particularly hydrogen bonds with Arg111 and Phe113, as well as significant interactions within the PTP domain involving Glu250, essential for high-affinity binding. To validate the accuracy of our docking program, we re-docked SHP099 to the PTPN11 active site (Fig. 5a). The re-docked conformations of the reference inhibitor SHP099 were meticulously compared with its original conformation in the co-crystallized structure. Remarkably, the Root Mean Square Deviation (RMSD) was found to be 0.02 Å, affirming the robustness of our docking algorithm and the reliability of our subsequent analysis. The docking score obtained for SHP099 was − 6.865, providing a benchmark for our interaction studies.

In the aqueous extract, the metabolite 3,3′,4′,5,6,7,8-Heptahydroxyflavone (PubChem CID: 44260065) exhibited a notable binding affinity, establishing hydrogen bonds at key residues such as THR:218, GLU:110, ARG:111, GLY:115, GLU:250, and GLU:249, with a docking score of − 8.901 and a Glide gScore of − 8.954 (Fig. 5b). These interactions mirror those observed with SHP099, especially at ARG:111 and GLU:250. Furthermore, other high-affinity binding partners from the aqueous extract include NADH (PubChem CID: 928), Succinyladenosine (PubChem CID: 20849086), DL-Dopa (PubChem CID: 836), and Xanthohumol B (PubChem CID: 9799506) (Fig. 5c–f). From the ethanolic extract, metabolites such as Alfafuran (PubChem CID: 90703276), (9S,10S)-9,10-dihydroxyoctadecanoate (PubChem CID: 12235230), Dihydromyricetin (PubChem CID: 25244375), LysoPE (PubChem CID: 53480953), and ADP-ribose 1"–2" cyclic phosphate (PubChem CID: 53481021) exhibited robust binding affinity towards the PTPN11 active site (Fig. 5 g–k). Notably, Dihydromyricetin showcased a docking score of − 7.987 and a Glide gScore of − 8.022, forming hydrogen bonds at key residues akin to those of SHP099.

The detailed docking scores, interaction patterns, and Glide scores for these interactions, as presented in Supplementary Tables S11 and S12, highlight the potential functional roles of these metabolites in regulating wound healing and coagulation cascades. The alignment of interaction sites with SHP099 and the comprehensive docking scores for both aqueous and ethanolic extracts underscore the predictive accuracy of our molecular docking analysis. This foundational work paves the way for further experimental validation and opens promising avenues for drug development in wound healing and coagulation cascade regulation.

### MRM-based validation of *H. alternata* metabolites with wound healing potential

**Figure 6:**
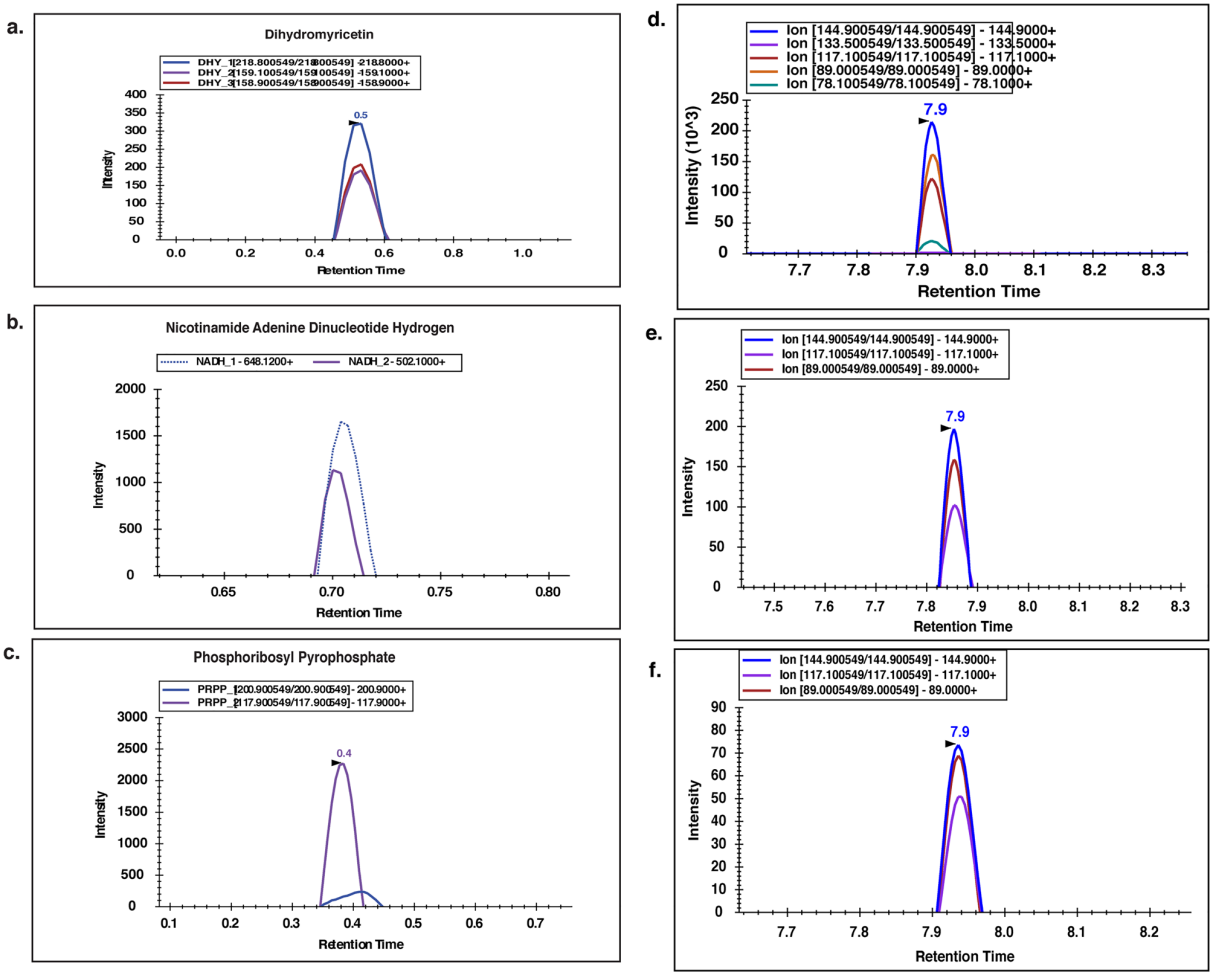
Targeted LC–MS/MS MRM validation for (**a**) Dihydromyricetin, (**b**) Nicotinamide adenine dinucleotide hydrogen, (**c**) phosphoribosyl pyrophosphate. Targeted LC–MS/MS MRM validation of curcumin. (**d**) Targeted LC–MS/MS MRM spectra of standard curcumin acquired in positive polarity, (**e**) targeted LC–MS/MS MRM transition spectra of curcumin from aqueous extract of *H. alternata* leaf, (**f**) targeted LC–MS/MS MRM transition spectra of curcumin from ethanolic extract of *H. alternata* leaf. The MRM transitions as provided in the legend.

Docking results show that the *H. alternata* metabolites such as NADH, Phosphoribosyl pyrophosphate (PRPP), and Dihydromyricetin (DHY) are the potential compounds directly interacting with key proteins involved in wound healing. So, these metabolites were further validated using MRM analysis. The targeted analysis was carried out for all three metabolites in positive polarity. Using the manual tuning method, the precursor-fragment masses were obtained and 2 to 3 transitions were identified for these metabolites, which were matching to their experimental or predicted spectra available on Competitive Fragmentation Modelling for Metabolite Identification (CFM-ID) and HMDB (Fig. 6a–c). The solvent gradients used for each MRM analysis is provided in Supplementary Table S13. Table 1 lists the precursor mass, transitions, and retention time along with the mass spectrometer parameters-declustering potential (DP), entrance potential (EP), collision energy (CE) and collision cell exit potential (CXP) of NADH, Phosphoribosyl pyrophosphate (PRPP), and Dihydromyricetin (DHY).

**Table 1:** The precursor mass, transitions and polarity of NADH, PRPP, and DHY along with the optimized parameters.

Metabolites	Q1	Q3	Time (ms)	Retention time (min)	DP	EP	CE	CXP
NADH	666.0	648.12; 502.10	50	0.7	33.68	10.0	12.86; 32.53	10.0
PRPP	391.0	200.90; 117.90	50	0.4	20.69	11.36; 14.16	43.43; 17.93	32.43; 17.93
DHY	321.0	218.8; 159.1; 158.9	50	0.5	246; 251; 246	10.0	27.0; 37.0; 37.0	26.0; 14.0; 18.0

Moreover, we identified the presence of curcuminoids such as complex curcumin and curcumin II in *H. alternata* leaf extract at the MS/MS level. So, MRM was performed for the detection of curcumin in *H. alternata* leaf extract samples with reference to standard curcumin. The sample was analyzed in technical triplicates. Five MRM transitions were obtained for the curcumin standard (Fig. 6d). The transitions were obtained in positive polarity with a precursor mass of 369 Da for curcumin from *H. alternata* sample. A total of three transitions were observed in both aqueous (Fig. 6e) and ethanolic extracts (Fig. 6f) of *H. alternata* sample in MRM mode. The transitions observed were 89 Da, 117.1 Da, 144.9 Da. Curcumin in aqueous extract was quantified by generating a calibration curve with concentrations ranging from 3.4 ng to 875 ng. (Supplementary Fig. S2) The amount of curcumin was found to be 32 ngs per milligram of the leaf extract. The declustering potential (DP), entrance potential (EP), collision energy (CE) and collision cell exit potential (CXP) were optimized for each of the transitions individually as provided in Table 2.

**Table 2:** The precursor and product mass of curcumin in positive polarity along with optimized parameters.

Q1	Q3	Time (ms)	ID	DP	EP	CE	CXP
396	89	50	Cur_1	23	10	83	10
396	117.1	50	Cur_2	96	10	62	10
396	144.9	50	Cur_3	44.1	10	41	10

## Discussion

Plant metabolites are known to promote wound healing due to their astringent and antimicrobial properties, which appear to be responsible for wound contraction and elevated epithelialization^31,32^. The *H. alternata* is a known wound healer that is used to treat wounds, and cuts and also directly apply the plant juice on wounds to prevent bleeding^4,33^. The topical application of *H. alternata* leaf paste promotes wound healing in mice by enhancing wound contraction and epithelialization^34^. Previous research has shown that the hexane extract of *H. alternata* has shown significant biological activity, including anti-bacterial, anti-oxidant, and anti-elastase activity^35^. However, the mechanism of action and molecular functions of *H. alternata* metabolites in wound healing is not yet identified. The global and targeted metabolomics approach using mass spectrometry has the potential to detect and characterize metabolites^36^.

The current research aims to explore the active components and constituents of *H. alternata* through liquid chromatography-mass spectrometry and network pharmacology tools to discover molecular networks associated with the wound-healing process. LC–MS/MS analysis identified several flavonoids, sesterterpene, alkaloids, ketones, diterpenes, sesquiterpenes, and flavonol in *H. alternata.* Flavonoids and alkaloids are shown to have anti-oxidant, anti-bacterial anti-inflammatory activities, anti-fungal, anti-viral and analgesic properties^37,38^. This study identified various terpenes, which are known for their wide range of medicinal properties including, anti-microbial, anti-inflammatory effect, wound healing, improved blood circulation, and application in treating conditions such as malaria, bacterial infection and migraines^39,40^. This study highlights the presence of several phytochemicals in *H. alternata* such as carboxylic acids, terpenoids, steroids and steroid derivatives, amino acids, peptides, carbohydrates and carbohydrate conjugates, which were previously reported^41,42^. We evaluated the biofunctions of *H. alternata* metabolites and the results showed the enrichment of components of various amino acids (serine, threonine, proline, arginine and glycine) metabolism and biosynthesis. Extensive research has been conducted on the involvement of amino acid metabolism in the process of wound healing^43–45^. In addition, we identified metabolites involved in vasoconstriction and vasodilation, crucial processes in the initial phase of wound healing (Fig. 1c). The metabolite pathway mapping indicated significant enrichment of pathways associated with amino acid metabolism (D-glutamine and D-glutamate metabolism and arginine metabolism), biosynthesis of glycosphingo lipid, CoA and terpenoid backbone and citrate cycle (TCA) (Fig. 2a). Studies has demonstrated that the plasma concentration of arginine and glutamine decreases during conditions such as injury or wound. These amino acids are essential for various physiological processes, including immune function and tissue repair^46^.

To gain more insights, *H. alternata* metabolites were subjected to network pharmacology analysis. This allowed us to identify the target proteins of *H. alternata* metabolites, deciphering their molecular action. The interaction between metabolites and proteins is crucial for regulating protein functions, consequently influencing cellular processes^47,48^. Pathway analysis results imply that *H. alternata* metabolites might influence all stages of wound healing such as hemostasis, inflammation, proliferation and remoldeling^46^ by interacting with key proteins associated with these processes. Hemostasis, the initial stage of wound healing is characterized by platelet aggregation and fibrin clot formation^46^. Our results indicate the involvement of *H. alternata* target proteins in pathways of platelet activation, signaling and aggregation, platelet aggregation, formation of fibrin clots and plug formation. This inference reinforces the potential role of *H. alternata* extract in halting bleeding. The common proteins involved in these pathways are *ABCC4, ADRA2A, ADARA2C, ADRA2B, F2, PTPN11,* and *YWHAZ*. Many reports have demonstrated that B2-adrenoceptors (beta2-ARs) such as *ADRA2A, ADARA2C,* and *ADRA2B* are thought to contribute to the modulation of pain regulation of blood flow by vasoconstriction and vasodilation of blood vessels^49,50^. It can also influence inflammation, which is a critical component of the early stages of wound healing^51^. Zhao et al.^52^ found that the expression of *ADRA2A* regulates the expression and secretion of growth factors, cAMP content, and activity of PKA in adipose-derived stem cells in type 2 diabetic (T2D) mice and subsequently influences wound healing. In addition, increased expression of *ADRA2A* and *ADARA2B* has been associated with decreased cAMP production leading to impaired re-epithelialization and granulation tissue formation in chronic wounds^53^. F2 (prothrombin) is a key coagulation factor, in active form (thrombin) it converts fibrinogen to fibrin to form a grid to trap red blood cells and platelets. Thrombin is also involved in inflammation, tissue re-modelling and healing^54^. Previous reports have shown that the protein encoded by the PTPN11 (Protein Tyrosine Phosphatase, Non-Receptor Type 11) gene, SHP2 is essential for differentiation, proliferation, metabolism in osteoblasts and epithelial to mesenchymal transition^55,56^. Furthermore, an in-vivo study reported that SHP2 activates HIF-1α by inhibiting 26S proteasome activity and promoting revascularization of wounds^57^. Moreover, YWHAZ (Tyrosine 3-Monooxygenase/Tryptophan 5-Monooxygenase Activation Protein, Zeta) is known to be involved in signal transduction and regulates a variety of cellular processes including cell division and the stress response. In wound healing, it could be implicated in cell signaling pathways that control cell proliferation, migration, and survival, all of which are vital for effective tissue repair^58^.

*H. alternate* metabolites may influence inflammation, the second stage of wound healing by interacting with proteins associated with the immune system and interferone signaling (CA1, UBE2N, RORC, PTPN11, PKM and CASP2). The inflammatory response promotes the recruitment of immune cells such as neutrophils, macrophages, dendritic cells and lymphocytes to clear the invading pathogens and also activate the secretion of several proteins that help heal wounds^59^. The pathway analysis also showed significant enrichment of pathways associated with cell proliferation and tissue remodelling such as PI3K cascade, MAPK signaling and RUNX1 regulating transcription of genes involved in differentiation of keratinocytes and signaling by FGFR1/2/3. The proliferative phase of wound healing is characterized by the proliferation, migration and differentiation of keratinocytes. PI3K signaling activates several downstream molecules including AKT, mTOR and GSK3and promotes the expression of VEGF, cyclin D1, and c-My which are essential for cellular proliferation, angiogenesis, metabolism and cellular survival^60^. MAPK signaling and signaling mediated by the RUNX1 are mainly involved in the differentiation and migration of keratinocytes and result in complete epidermal regeneration^61^. In-vivo study reported the role of MAPK signaling in the proliferation and migration of keratinocytes under skin damage^62^.

In-silico docking results show that several metabolites have potential interaction ability with key proteins involved in wound healing such as *F2* and *PTPN11*. Prior to molecular docking, the reference inhibitors and our plant metabolites were prepared using the 'Ligprep' settings in GLIDEv7, optimizing their molecular structures for the subsequent docking process. To evaluate the effectiveness of protein–ligand binding, we utilized an extra precision (XP) scoring function to flexibly dock the compounds within the active sites of F2 and PTNPN11^63^. We further validated the docking methodology by performing re-docking of reference inhibitors of PTPN11 and obtained the RMSD of 0.02 Å affirming the robustness of our docking algorithm. The metabolites such as NADH and phosphoribosyl pyrophosphate (PRPP), an important intermediate in cellular metabolism^64^ showed higher docking scores with F2, the protein involved in the coagulation cascade. PTPN11 showed higher docking affinity with flavonoids such as 3,3′,4′,5,6,7,8-Heptahydroxyflavone and dihydromyricetin. Recent studies demonstrated that hydroxyflavone may play a potential role in preventing inflammation^65,66^. Furthermore, dihydromyricetin participates in diverse functions such as anti-inflammatory and antioxidant activities, enhancement of mitochondrial function, and the regulation of autophagy^67,68^. These findings, encapsulating a blend of detailed docking analysis shed light on the promising potential of *H. alternata* metabolites as therapeutic agents in the coagulation cascade, particularly for wound healing. Nonetheless, it's important to acknowledge that these results are preliminary and predictive, emphasizing the necessity for further experimental studies to verify the biological activity and therapeutic potential of these metabolites.

Among the metabolites identified from in-silico docking, NADH, Phosphoribosyl pyrophosphate, and Dihydromyricetin were further validated by using targeted MRM analysis. Known transitions of these metabolites were successfully detected by the MRM method. NADH is a predominant coenzyme, which plays a major role in the generation of energy, metabolism, reduction–oxidation (redox) reactions, cell survival and death. It is also known to be involved in the regeneration of epithelium during wound healing^69^. Phosphoribosyl diphosphate is an important intermediate in cellular metabolism and an essential component for the synthesis of purine and pyrimidine nucleotides^64^. Dihydromyricetin is a flavonoid with various properties such as antioxidative, anti-inflammatory, anticancer, antimicrobial, and lipid and glucose metabolism-regulatory activities^70^.

The global metabolomic profiling of *H. alternate* plant extract detected the presence of curcuminoids such as complex curcumin and curcumin II, the major turmeric spice polyphenols. Curcumin is known to be involved in several cellular pathways including proinflammatory cytokines, apoptosis, NF–κB, transforming growth factor-β, and STAT3 pathways^71^. Curcuminoids have been shown to participate in the wound healing process by enhancing fibroblast proliferation, granulation tissue formation, and collagen deposition^24,72–74^. The presence of curcumin in *H. alternata* was further confirmed by targeted MRM validation and metabolite quantification. MRM analysis detected three transitions of curcumin in the aqueous and ethanolic extract of *H. alternata* in positive mode.

## Conclusions

The global metabolite profile of *H. alternata* was performed to investigate the metabolites involved in wound healing. The *H. alternata* metabolites interacting with F2 and PTPN11 proteins may hold functional imperatives in lead validation, lead optimization, and mechanistic insights for drug development. *H. alternata* is a promising wound-healing promoter worthy of further studies and clinical evaluation. Thus, the present study confirms the potential value of *H. alternata* by the presence of various compounds. Given the prominence of metabolite and protein interactions discovered in this study, further investigation of metabolite signatures related to the wound healing process would help to identify and manipulate the key molecules involved in wound healing. A combination of metabolites involved in wound healing identified in this study, as a cocktail may have a future application in wound therapy, which can be tested by in-vivo studies and clinical trials. Further investigation will be needed to elucidate the role of metabolites in wound healing.

## Materials and methods

### Plant material collection and metabolite extraction

*H. alternata* (*Strobilanthus alternata*) is grown as a ornamental plant. We collected the leaves of this plant from the cultivated gardens of Yenepoya (Deemed to be) University. Dr. TSKP (Author in this manuscript) has identified it. The identification was also confirmed by Dr. K. R. Chandrashekhar, ex-Professor in the Department of Applied Botany, Mangalore University, India. National Biodiversity Act of 2023, under the Government of India allows the use of any plants for research by Indian citzens. Hence, use of *H. alternata* (either cultivated or wild), including the collection of plant material, complies with relevant institutional, national, and international guidelines and legislation. The dirt on the leaves was washed off and allowed to dry. Dry leaves were then crumbled into fine powder. The aqueous and ethanolic extracts were prepared by adding distilled water and absolute ethanol (Ratio-1:4) to the leaf powder, respectively, and kept for maceration at 37 °C at 120 rpm for 3 days. Then, the mixture was filtered using filter paper, and the supernatant was concentrated using an oven to get the crude extract.

### LC–MS/MS analysis of *H. alternata*

An aliquot of ethanolic and aqueous extract residue of *H. alternata* was dissolved in 1 mL of 70% methanol to prepare a final concentration of 1 mg/mL and centrifuged at 10,000 rpm for 15 min. The collected supernatant underwent LC–MS/MS analysis employing a QTRAP-6500 mass spectrometer (AB SCIEX, USA) coupled with an Agilent Infinity II 1290 liquid chromatography system (Agilent Technologies, Inc., Santa Clara, CA, USA), following the method outlined in Saraf et al. (2020) with specific modifications. The separations were carried out using the ZORBAX Eclipse plus C18, RRHD (rapid resolution high definition) reverse phase column (2.1 × 150 mm, 1.8 µm). In each analysis, 10 µL of the sample was injected into the chromatography column and eluted at 35 °C with a flow rate of 0.25 mL/min with a 40-min gradient with the mobile phase (polar solvent A—0.1% formic acid and organic solvent B—90% acetonitrile in 0.1% formic acid). The gradient used for elution consists of 2% solvent B (0–1 min), 30% solvent B (1–8 min), 60% solvent B (8–16 min), 98% solvent B (16–24 min), 98% solvent B (24–32 min), 2% solvent B (32–36 min) and 2% solvent B (36–40 min).

Mass spectra for aqueous and ethanolic extract of *H. alternata* were acquired in triplicates. The MS data was acquired through independent positive and negative modes with a mass range from 50 to 1000 and functions based on the EMS-IDA-EPI (enhanced mass spectra-independent data acquisition-enhanced product ion) method. In the MS1 scan, only the top five ions were selected based on their intensity and were further fragmented at the MS/MS level. Electrospray ionization (ESI), Turbo Spray Ion Drive was used as a source. The source temperature was set at 450 °C; ion source voltages were 4500 V in the positive mode − 4500 V in the negative mode; source gas I and gas II at 40 psi and curtain gas at 30 psi. The optimized declustering potentials for positive and negative modes were set at 75 V and − 75 V, respectively. The collision energy (CE) was set as 40 V and − 40 V in positive and negative modes.

### Data analysis

Metabolite data analysis was carried out at MS1 and MS/MS levels using MZmine^28^ and MS2Compound (https://sourceforge.net/projects/ms2compound/)^29^. We also carried out MS1 level analysis using XCMS Online (Forsberg et al*.,* 2018). MS data obtained in .wiff format was converted into .mzmL format using MSConvert, ProteoWizard software^75^. The .mzmL files were processed with MZmine 2.53 as described by Karthikkeyan et al. (2022). The analysis includes the following steps, such as data file import, mass detection, peak picking, peak extension, chromatogram deconvolution, isotope peak grouping, peak alignment across replicates/samples, gap filling, filtering of duplicate peaks and data export. The .mzmL files of aqueous and ethanolic extracts were imported to MZmine and feature detection was performed at MS1 and MS/MS levels. The centroid data with a noise level greater than 1.0E3 was set for MS1 level mass detection and greater than 1.0E1 was set for MS/MS level. The chromatographic peak with a minimum time range of 10 min for peak duration, the amplitude of noise at 1.5E2 a minimum peak height of 1.0E3 and an m/z tolerance of 5 parts per million were selected for chromatogram deconvolution. The chromatogram deconvolution and isotope peak grouping were performed with the noise amplitude and isotopic peak grouper algorithms. The relevant peaks across replicates were aligned with the Joinaligner. Gap filling and filtering of duplicate peaks was carried out with peak finder (multi-threaded) and duplicate peaks filter algorithms.

The metabolite features obtained from MZmine analysis were further used for compound detection at MS1 and MS/MS levels with an in-house built MS2Compound^29^. The feature I.D. and m/z values of precursor ions in .text format were uploaded as an input file for MS1 level analysis, and the .mgf files obtained from MZmine analysis containing feature-I.D., precursor ion mass, scan number, retention time, charge and fragment ion masses were used as an input for MS/MS level compound identification. The following parameters were selected in MS2Compund for MS1 and MS/MS level analysis. The databases chosen for compound identification include PlantCyc^76^, Kyoto Encyclopedia of Genes and Genomes (KEGG) (www.genome.jp/kegg/compound/) and Phenol-Explorer^77^ and Human Metabolome Database (HMDB)^78^ precursor tolerance was set as 0.05 Da. The fragment tolerance of 0.5 Da and minimum fragment match of 2 was selected for the MS/MS level search. The assigned, non-redundant metabolites with rank 1 were chosen for downstream bioinformatics analysis.

Metabolite identification at the MS1 level was also carried out by XCMS Online analysis to improve the identification. The .mzmL files were uploaded through a Java applet, and parameters that matched the instrument setup were selected. The monoisotopic mass obtained from XCMS Online analysis was searched in HMDB for metabolite assignment. The assigned metabolites with the lowest delta ppm value were selected for downstream analysis.

### Bioinformatics analysis

The non-redundant metabolites identified in aqueous and ethanolic were analyzed for metabolite classification and biofunction by using MBROLE version 2.0 and pathway enrichment analysis was performed by using MetaboAnalyst 5.0 tool^30^; Pang et al*.,* 2021). Those with a *p*-value less than 0.05 were considered significant.

BindingDB analysis was carried out to determine the protein targets of metabolites, the compound names of identified metabolites at the MS/MS level were converted into their SMILES IDs using the PubChem Identifier Exchange Service (https://www.pubchem.ncbi.nlm.nih.gov/idexchange/idexchange.cgi)^79^. The SMILES IDs of metabolites were used as inputs for the BindingDB analysis, which predicts the protein-metabolite interactions based on their structural similarity and previously known interactions. A similarity score cut-off of ≥ 0.85 was given to identify the protein targets interacting with the metabolites, and those hit with a score of 1.0 indicate an exact match. UniProt IDs were converted into official gene symbols using the Biological Database network (bioDBnet)^80^. The Gene Ontology analysis was conducted on protein targets of metabolites belonging to Homo sapiens, utilizing the Database for Annotation, Visualization and Integrated Discovery (DAVID) v6.8^81^ and pathway enrichment analysis was carried out using the Reactome pathway database (https://reactome.org/PathwayBrowser/)^82^.

### Molecular docking of targets of *H. alternata* metabolites

The molecular docking analysis was conducted using Glide v7.7, part of the Schrödinger suite (Schrodinger, LLC, New York, NY, 2019–1). To ascertain protein–ligand binding efficacy, we employed an extra precision (XP) scoring function for flexibly docking the compounds with the active sites of the F2/Prethrombin-2 complex (PDB ID: 3K65; Resolution: 1.85 Å) and PTPN11 (PDB ID: 5EHR; Resolution: 1.70 Å). Our procedure entailed several crucial steps: protein preparation, ligand preparation, grid generation, and molecular docking.

Initially, we obtained the three-dimensional crystal structures of the F2/Prethrombin-2 complex and PTPN11 from the Protein Data Bank. For the F2/Prethrombin-2 complex, lacking known inhibitors, we utilized SiteMap for active site prediction. In contrast, for PTPN11, we selected the structure 5EHR, complexed with the potent inhibitor SHP099. SiteMap, a tool within the Schrödinger suite, was instrumental in identifying and analysing potential active sites on the F2/Prethrombin-2 complex, aiding in understanding interaction dynamics and preparing for accurate docking simulations. Before molecular docking, both the reference inhibitors and our plant metabolites underwent preparation using the GLIDEv7 'Ligprep' settings, optimizing the molecular structures for the docking process. We then docked the ligands to the binding sites of the F2/Prethrombin-2 complex and PTPN11 using the Extra Precision (XP) docking algorithm, focusing on assessing binding affinities and interaction patterns.

To validate the accuracy of our docking methodology, a re-docking procedure was performed for the reference inhibitors of PTPN11. The re-docked conformations were rigorously compared with their original conformations in the co-crystallized structures, ensuring the robustness of our docking algorithm and the reliability of our subsequent analyses. The docking scores, G scores, and the nature of the protein–ligand interactions were meticulously reported for the top ligands, providing insights into their binding efficacy and potential as therapeutic agents.

### MRM-based validation of *H. alternata* metabolites with wound healing potential

A stock solution of 1 mg/mL of curcumin (CRMN) from HiMEDIA (RM1449) was prepared with DMSO and stored at − 20 °C. The solvents used were acetonitrile (ACN) of LCMS grade procured from Millipore, water (Milli Q) and formic acid of analytical grade from Sigma.

LC–MS/MS analysis was carried out using a QTRAP 6500 mass spectrometer (SCIEX, Framingham, MA, USA) interfaced with a 1290 Infinity HPLC system (Agilent Technologies). A programmed autosampler injected the samples onto the Zorbax RHP column with the dimensions of 2.1 mm x 50 mm, 2.7 μm (Agilent Technologies, USA). The mobile phases used for the analysis were 0.1% formic acid in water as solvent A and 0.1% formic acid in 90% acetonitrile as solvent B and the flow rate was set to 0.300 mL/min. The total run time was 15 min, and 15 μL of the sample was injected into the column. Data were acquired in positive mode using the MRM scan mode. The Analyst software (version 1.6.3) was used to acquire data, and the Mass range was 50–500 Da. Samples were ionized using the ESI source, gasses were maintained as Gas 1 (GS1) at 35 psi, Ion Source Gas 2 (GS2) at 35 psi, Curtain gas (CUR) at 20.0 psi, ESI source temperature was maintained at 450 °C and collision activated dissociation (CAD) gas at high. The ion spray voltage was set to 5500 V. Analysis of the target metabolites was carried out by using Skyline software (version 21.2.0.425, 64-bit). Metabolite transitions were provided as input for the analysis. Based on the retention time, and peak intensity, the peaks were picked from the raw file.

### Supplementary Information

Supplementary Information.

## Data Availability

The mass spectrometry data have been submitted to MassIVE—Mass Spectrometry Interactive Virtual Environment https (https://massive.ucsd.edu), with the study identifier MSV000088024.
